# Notes on the Treatment of Amebic Dysentery with Emetine and Bismuth Iodide

**Published:** 1919-01

**Authors:** 


**Notes on the Treatment of Amebic Dysentery with Emetine and Bismuth Iodide.** By A. C. Lambert. *Journal of the Royal Army Medical Corps*, July, 1918.

An average of 29% of the cases of amebic dysentery, admitted in July and August, 1917, to hospitals in Macedonia, were treated with emetine and bismuth iodide; sometimes connectively with emetine hydrochloride. All these cases were severe, some fatal. The patients were natives from all parts of India, and they tolerated the drug very well; — a fact to be taken into account.

The following facts deserve attention :

1. The tendency or non-tendency of the drug to cause vomiting.

2. Its action, either when given alone, or in conjunction with emetine hydrochloride in acute cases, less acute cases, or in chronic relapsing cases. In no way were more than 3 grains given at a time, nor more than four in 24 hours. Vomiting was easily checked by absorption of 15 drops of tinct. opii.

In acute cases, the most satisfactory method was to use 2 grains of emetine and bismuth iodide in pill form at night — after a feed of milk — and one grain of emetine hydrochloride hypodermically in the morning. Using the two drugs connectively brought a much more rapid and more complete recovery than the use of emetine alone. After an incredibly short treatment, mucus and blood were no longer found in the stools; the number of ameboid forms diminished after 24 hours; and disappeared altogether after a week. In one case griping and tenesmus were relieved after 24 hours.

In subacute cases good results were obtained by giving 2 grains of emetine and bismuth iodide in pills morning and evening, with an occasional dose of 1/2 ounce of sulphate of soda. Mucus and blood yielded rapidly to this treatment, which was discontinued after a total of 36 or 40 grains of emetine and bismuth iodide had been absorbed. Milk diet was given chiefly.

In chronic relapsing cases patients had to be treated as quickly as possible with emetine in order to destroy the ameboid forms endangering so gravely the coats of the bowel. The cases brought to the hospitals were very debilitated and anemic; emaciation was extreme, as may easily be understood, with patients passing from sixteen to twenty stools in a day, consisting of blood, mucus and sloughs, or even blood alone. In fatal cases the postmortems showed extensive ulceration and gangrene of the whole of the large bowel.

In the treatment, one grain emetine hydrochloride was given at first hypodermically twice daily; afterwards, with amelioration, one or two grains of emetine and bismuth iodide were substituted for one of the doses of emetine. Small doses of morphia were used to relieve griping and tenesmus and promote sleep. Enemata was not employed and rightly, as the autopsies proved, the bowels being too damaged to endure the drug without danger.

Convalescence in these cases was always very long, and the diet had to be appropriately composed, — nourishing and easily assimilable milk diet to which were added eggs, meat essences, light soups and stimulants, brandy or port wine.

If bismuth iodide used connectively with emetine has given splendid results, it should not, for that reason, be taken as a substitute for emetine.

